# *Tenebrio molitor* Proteins-Derived DPP-4 Inhibitory Peptides: Preparation, Identification, and Molecular Binding Mechanism

**DOI:** 10.3390/foods11223626

**Published:** 2022-11-13

**Authors:** Jiao Tan, Jing Yang, Xinyi Zhou, Ahmed Mahmoud Hamdy, Xilu Zhang, Huayi Suo, Yu Zhang, Ning Li, Jiajia Song

**Affiliations:** 1 College of Food Science, Southwest University, Chongqing 400715, China; 2 Chongqing Key Laboratory of Speciality Food Co-Built by Sichuan and Chongqing, Chongqing 400715, China; 3 Chongqing Engineering Research Center for Processing & Storage of Distinct Agricultural Products, Chongqing Technology and Business University, Chongqing 400067, China; 4 Westa College, Southwest University, Chongqing 400715, China; 5 Dairy Science Department, Faculty of Agriculture, Assiut University, Assiut 71526, Egypt

**Keywords:** edible insects, dipeptidyl peptidase-4, peptides identification, molecular docking, enzymatic hydrolysis

## Abstract

Inhibition of dipeptidyl peptidase-4 (DPP-4) is an effective way to control blood glucose in diabetic patients. *Tenebrio (T.) molitor* is an edible insect containing abundant protein. *T. molitor* protein-derived peptides can suppress the DPP-4 activity. However, the amino acid sequence and binding mechanism of these DPP-4 inhibitory peptides remain unclear. This study used the flavourzyme for *T. molitor* protein hydrolysis, identified the released peptides with DPP-4 inhibitory effect, and investigated the binding interactions of these peptides with DPP-4. The results showed that flavourzyme efficiently hydrolyzed the *T. molitor* protein, as demonstrated by the high degree of hydrolysis, disappearance of protein bands in SDS-PAGE, and changes to protein structure. The 4-h flavourzyme hydrolysates showed a good inhibitory effect on DPP-4 (IC_50_ value of 1.64 mg/mL). The fragment of 1000–3000 Da accounted for 10.39% of the total peptides, but showed the strongest inhibitory effect on DPP-4. The peptides LPDQWDWR and APPDGGFWEWGD were identified from this fraction, and their IC_50_ values against DPP-4 were 0.15 and 1.03 mg/mL, respectively. Molecular docking showed that these two peptides interacted with the DPP-4 active site via hydrogen bonding, hydrophobic interactions, salt bridge formation, π-cation interactions, and π-π stacking. Our findings indicated that *T. molitor* protein-derived peptides could be used as natural DPP-4 inhibitors.

## 1. Introduction

The prevalence of diabetes has been dramatically increasing worldwide in recent years. There are approximately 537 million adults with diabetes worldwide, and 6.7 million deaths among those with diabetes in 2021 (statistics from International Diabetes Federation). In fact, diabetes can lead to many macro- and microvascular complications and affect multiple organs, which seriously threatens human health and causes a huge burden to society [1,2]. Some treatment strategies for diabetes, including oral agents and subcutaneous injections of insulin, are applied to regulate fasting blood glucose in diabetic patients. Among these strategies, the use of dipeptidyl peptidase-4 (DPP-4) inhibitors is receiving wider attention due to its advantage of low risk of hypoglycemia [3,4]. After food intake, insulin secretion from pancreatic islets is induced by the glucose inhibitory polypeptide (GIP) and glucagon-like peptide-1 (GLP-1) [5,6], but these incretins can be inactivated by the metabolic enzyme DPP-4. Thus, inhibiting DPP-4 extends the half-life of active incretins, thereby contributing to glycemic regulation. Currently, there are some synthetic DPP-4 inhibitors available, but they may have side effects, including joint pain, inflammation of the pancreas, and allergic reactions. Thus, some natural functional components, such as protein peptides, resveratrol, and flavones have been screened for their inhibitory activity against DPP-4 [7,8]. Food proteins-derived bioactive peptides usually consisting of 2–20 amino acid residues have been reported to possess various health-promoting attributes, such as anti-hypertensive, anti-diabetic, cholesterol-lowering, immunoregulatory, anti-oxidant, and mineral absorption-promoting effects [9,10,11,12]. The peptides with inhibitory activity against DPP-4 have received increasing attention due to their higher potency, selectivity, and safety [11,13]. For example, the peptide QLRDIVDK from *Sorghum bicolor* protein has a strong affinity to DPP-4, thereby showing a potent inhibition of DPP-4 [8]. Chinese black tea-derived peptide AGFAGDDAPR inhibits DPP-4 activity, contributing to the improvement of pancreatic function in diabetic mice [14].

Edible insects, such as *Tenebrio (T.) molitor*, *Bombyx mori*, *Locusta migratoria*, and *Blaptica dubia*, are abundant in protein. The insect protein is considered an alternative protein source because of its low environmental effect [15]. Moreover, insect protein is an excellent source of bioactive peptides. It has been reported that insect protein-derived peptides possess various bioactivities, such as anti-oxidant, anti-hypertensive, anti-cancer, anti-inflammatory, anti-diabetic, and anti-microbial activities [16]. *T. molitor* is an edible, highly nutritious insect due to its high content of fats, proteins, vitamins, and minerals, which is consumed in many countries and has been processed into various insect-based food products, e.g., frankfurters [17,18]. The enzymatic hydrolysates of *T. molitor* protein have been shown to have antithrombotic activity, inhibit α-glucosidase, protect AML12 cells from oxidative damage, and decrease the blood pressure of hypertensive rats [19,20,21,22]. Moreover, compared with unhydrolyzed *T. molitor* proteins, the cuticular protein hydrolysates exhibit higher inhibitory activity against DPP-4 [23]. Although researchers speculate that the peptide APVAH from *T. molitor* protein is a DPP-4-inhibitory peptide [24], to date, the amino acid sequence of *T. molitor* protein-derived peptides with an inhibitory effect on DPP-4 activity remains unclear. This study determined the inhibitory activity of *T. molitor* protein hydrolysates against DPP-4, and then identified the DPP-4 inhibitory peptides by LC-MS/MS and solid-phase synthesis. Furthermore, the interaction types between peptides and DPP-4 active site were investigated by molecular docking. This is the first study to identify the *T. molitor* protein-derived peptides with DPP-4 inhibition and characterize their binding mechanisms with DPP-4.

## 2. Materials and Methods

### 2.1. Materials

Fresh *T. molitor* were donated by Prof. Ning Li at Chongqing Technology and Business University, while 8-anilino-1-naphthalenesulfonic acid (ANS), trypsin (250 N.F.U/mg), flavourzyme (≥30,000 U/g), papain (≥800,000 U/g), and alcalase (≥200,000 U/g) were bought from Beijing Solarbio Science and Technology Co., Ltd. (Beijing, China). The o-phthaldialdehyde (OPA) was bought from Shanghai Bide Medical Technology Co., Ltd. (Shanghai, China). The acetonitrile, trifluoroacetic acid, and formic acid were chromatographic grade and were supplied by Chengdu Kelong Chemical Co., Ltd. (Chengdu, China). DPP-4 (≥4000 units/μg) and its substrate Gly-Pro-p-nitroanilide were bought from Millipore Sigma (Burlington, MA, USA).

### 2.2. The Enzymatic Hydrolysis of T. molitor Protein

The *T. molitor* protein was isolated according to a reported method [25]. Briefly, lyophilized and defatted *T. molitor* were crushed, and dissolved with deionized water (1/20, *w*/*v*). After regulating the pH value to 9.5 by 1 M NaOH solution and stirring for 1 h, the solution was centrifuged to collect the supernatant (4 °C, 3000 g, 15 min). The supernatant was regulated to pH 4.5, and further centrifuged to collect the pellet, which was then freeze-dried.

In this study, the proteases alcalase, flavourzyme, papain, trypsin were used for the hydrolysis of *T. molitor* proteins. Briefly, the lyophilized protein (5%, *w*/*v*) was resuspended in deionized water, and then the temperature and pH value of the solution were regulated to the optimal temperature and pH values for the corresponding enzyme (alcalase, 50 °C, pH 11.0; flavourzyme, 50 °C, pH 7.0; papain, 55 °C, pH 6.0; trypsin, 37 °C, pH 8.0). Afterward, the corresponding enzyme was added at a ratio of 5% (*w*/*w*), maintaining constant temperature and pH during hydrolysis process. The reaction solution was sampled at different hydrolysis times (0, 30, 60, 120, 180, 240, and 300 min), and ultimately heated at 85 °C for 20 min. The supernatant of the reaction solution was adjusted to neutral pH, and lyophilized for subsequent use.

### 2.3. Degree of Hydrolysis (DH) Assay

The OPA method was adopted for the assay of the DH. In brief, the OPA working solution was prepared according to a previous publication [26]. The 3 mL of OPA working solution was mixed with 400 μL of serine standard (0.1 mg/mL), sample solution (1 mg/mL), and deionized water, respectively. After 2 min, the optical density at 340 nm was recorded. The DH was calculated according to a previously described equation [26]. In addition, the reducing sodium dodecyl sulfate-polyacrylamide gel electrophoresis (SDS-PAGE) was performed to evaluate the hydrolysis of *T. molitor* proteins. Briefly, each sample was mixed with a 5 × loading buffer (Beyotime, Shanghai, China), and boiled for 5 min. Then, 10 μL of each sample was loaded on the gel (Beyotime), and after protein separation by SDS-PAGE, the gel was stained with Coomassie Blue (Beyotime).

### 2.4. Analysis of DPP-4 Inhibition Activity

The inhibition of each sample on DPP-4 was assayed according to a previously described method [27]. Briefly, 20 μL of each sample solution, 50 μL of Tris-HCl buffer (pH8.0, 100 mM), and 100 μL of substrate were mixed in a well of a 96-well plate, then incubated at 37 °C for 10 min. Afterward, the 30 μL of DPP-IV solution was added, then placed at 37 °C for 40 min incubation. Afterward, the optical density was recorded at 405 nm using a BioTek Synergy H1 microplate reader (BioTek, Winooski, VT, USA). The inhibitory effect of the samples on DPP-4 was calculated according to a previously described equation [27]. 

### 2.5. Structural Characterization

#### 2.5.1. Ultraviolet (UV) Absorption Spectroscopy

The *T. molitor* protein hydrolysates (0.01%, *w*/*v*) were added to deionized water and resuspended well. The UV spectra of the hydrolysates were recorded using a Nanovue Plus UV-visible spectrophotometer (GE Healthcare Life Science, Piscataway, NJ, USA) in the range of 190–800 nm.

#### 2.5.2. Relative Fluorescence Intensity

A 1.5 mL aliquot of *T. molitor* protein hydrolysate solution (1 μg/mL, in 10 mM PBS at pH 7.0) was mixed with 20 μL of an 8 mM ANS solution. Relative fluorescence intensity was assayed using an F-2500 fluorescence spectrometer (Hitachi Ltd., Tokyo, Japan) in the range of 400–700 nm [28].

#### 2.5.3. Circular Dichroism (CD) Spectroscopy

After resuspending the *T. molitor* protein hydrolysate (0.01%, *w*/*v*) in deionized water, the secondary structure of the protein peptides was analyzed by a MOS-500 CD spectrometer (Bio-Logic, Seyssinet-Pariset, France) in the range of 190–250 nm. The scan speed was 100 nm/min, and the path length was 1 mm. The ellipticity (θ) was measured in millidegrees (mdeg).

#### 2.5.4. Molecular Weight Distribution

A TSK^®^ gel G2000 SWXL (7.8 mm × 300 mm) column (Tosoh, Tokyo, Japan) was used for separation. The mobile phase consisted of acetonitrile, water, and trifluoroacetic acid (40/60/0.05, *v*/*v*/*v*). The flow rate was 0.5 mL/min, and monitoring wavelength was 214 nm [29].

### 2.6. Peptide Identification

The peptides of the *T. molitor* protein hydrolysates were analyzed by LC-MS/MS [30]. Briefly, the peptides were separated on a U-3000 ultra-high performance liquid chromatography system (Thermo Fisher Scientific Inc., Waltham, MA, USA) coupled with an Orbitrap Fusion™ Tribrid™ mass spectrometer (Thermo Fisher Scientific Inc.), equipped with a C18 (4.6 mm × 250 mm, 5 μm, 300 Å) column (Grace Vydac, Hesperia, CA, USA). The mobile phases used for peptide separation consisted of 0.1% formic acid (FA) in acetonitrile and 0.1% FA in water. The recorded MS/MS spectra were searched in the UniProt database using the SEQUEST Proteome Discoverer 1.4 package (Thermo Fisher Scientific Inc.). PeptideRanker (http://distilldeep.ucd.ie/PeptideRanker/) (accessed on 21 October 2022) and ToxinPred (https://webs.iiitd.edu.in/raghava/toxinpred/design.php) (accessed on 21 October 2022) were used to predict the possible bioactivity and toxicity of the peptides.

### 2.7. The Synthesis of Peptide

The conventional Fmoc solid-phase synthesis method was used to prepare peptides with a purity of ≥ 95%, which was performed by Guotai Biotechnology Co. Ltd. (Hefei, China).

### 2.8. Molecular Docking

The three-dimensional (3D) structure of human DPP-4 (2ONC) was downloaded from the Protein Data Bank. The two-dimensional (2D) structure of the synthesized peptide was drawn using ChemDraw 19.0 (PerkinElmer, Waltham, MA, USA), and then converted to 3D structure and subjected to energy minimization with the Chem3D 19.0 software (PerkinElmer, Waltham, MA, USA). The University of California, San Francisco (UCSF) DOCK 6.9 (https://dock.compbio.ucsf.edu/) (accessed on 21 October 2022) was used to predict the binding between the synthesized peptides and the DPP-4 enzyme. The flexible ligand docking was executed and the grid score (kcal/mol) was calculated for each output pose.

### 2.9. Statistical Analysis

The data is shown as the mean ± standard deviation. The one-way analysis of variance (ANOVA) and Tukey′s post hoc test were used for the statistical analysis (*p* < 0.05, significant differences).

## 3. Results

### 3.1. DH of the T. molitor Protein Hydrolysates

**Figure 1:**
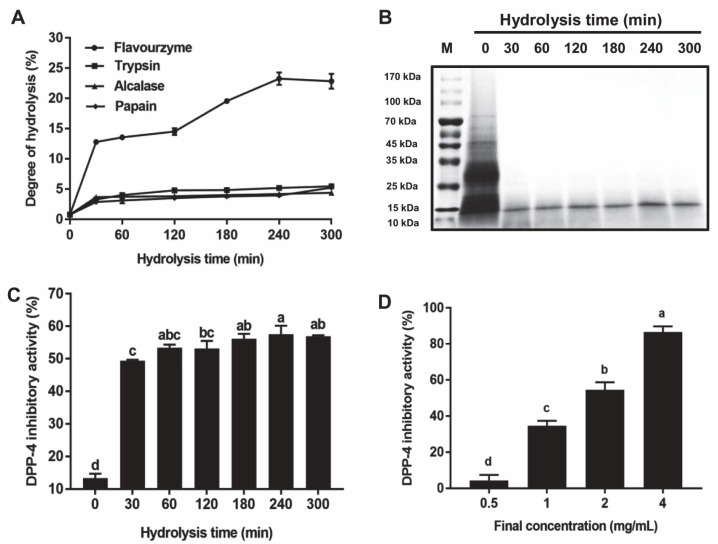
The degree of hydrolysis (DH) and inhibitory activity against dipeptidyl peptidase-4 (DPP-4) of *Tenebrio (T.) molitor* protein hydrolysates. (**A**) DH of *T. molitor* protein hydrolysates. (**B**) Sodium dodecyl sulfate-polyacrylamide gel electrophoresis (SDS-PAGE) analysis of *T. molitor* protein hydrolysates. (**C**) Inhibitory effect of *T. molitor* protein hydrolysates (at a final concentration of 2 mg/mL) on DPP-4 activity. (**D**) Inhibitory effect of 4-h flavourzyme hydrolysates (at different concentration) on DPP-4. Data are shown as the mean ± SD (*n* = 3), and different letters mean significant difference (*p* < 0.05).

The results shown in Figure 1A reveal that the DH of all the hydrolysates generated by trypsin, alcalase, and papain was less than 6.5%, while that generated by flavourzyme hydrolysates was higher. The DH of the flavourzyme hydrolysates was increased with the reaction time, and the 4-h hydrolysates had the highest DH (23.25 ± 1.03%). The SDS-PAGE analysis indicated that the major bands of *T. molitor* protein hydrolysates distributed from 10 to 100 kDa, and flavourzyme treatment effectively hydrolyzed *T. molitor* protein above 15 kDa (Figure 1B).

Taken together, the flavourzyme hydrolysates had the highest DH in comparison to the hydrolysates generated from other proteases, indicating that the flavourzyme hydrolysis contributed to the release of short-chain peptides from *T. molitor* protein. Generally, the short-chain peptides have great potential in exerting biological activities. Thus, the flavourzyme was selected for subsequent *T. molitor* protein hydrolysis.

### 3.2. Inhibition Effect of the T. molitor Protein Hydrolysates on DPP-4

The results presented in Figure 1C show that the inhibitory activity of all flavourzyme hydrolysates against DPP-4 was higher compared with *T. molitor* protein (*p* < 0.05). Although the DPP-4 inhibitory effect of these hydrolysates increased little with the hydrolysis time, the 4 h enzymatic treatment reached the greatest inhibitory activity against DPP-4 (57.56 ± 2.59%) and thus was used for subsequent analysis. Furthermore, the 4-h flavourzyme hydrolysates showed a dose-dependent inhibitory effect on DPP-4 activity (Figure 1D), and its inhibitory activity against DPP-4 reached up to 86.59 ± 3.21% (final concentration of hydrolysates, 4 mg/mL).

### 3.3. Structural Characteristics of the T. molitor Protein Hydrolysates

**Figure 2:**
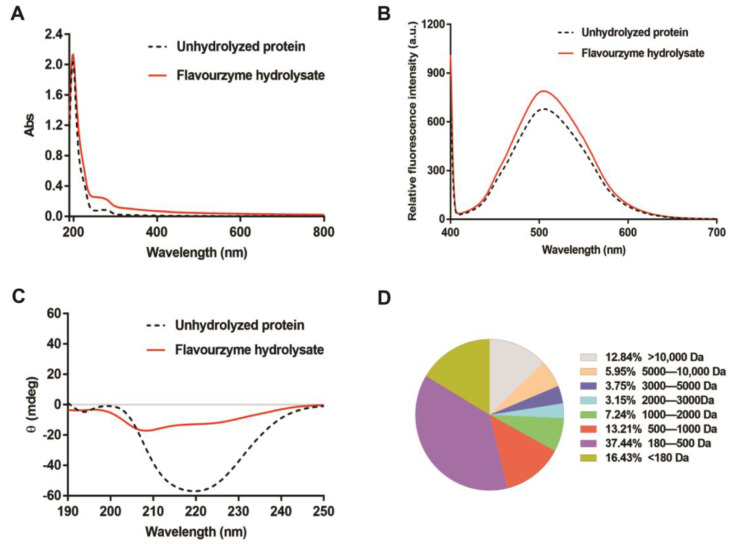
The structural characterization of *Tenebrio molitor* protein hydrolysates generated by flavourzyme hydrolysis for 4 h. (**A**) UV absorption spectroscopy. (**B**) Relative fluorescence intensity. (**C**) Circular dichroism spectroscopy. (**D**) Molecular weight distribution.

In comparison to the control sample (unhydrolyzed *T. molitor* protein), the UV absorption of flavourzyme hydrolysates is markedly increased, indicating that flavourzyme treatment led to conformational changes of the proteins and thereby the exposure of aromatic amino acids (Figure 2A). As we anticipated, the relative fluorescence intensity of the *T. molitor* protein hydrolysates after ANS binding was higher than that of unhydrolyzed *T. molitor* protein, demonstrating that the flavourzyme hydrolysis led to the exposure of hydrophobic residues (such as aromatic amino acids) of *T. molitor* proteins (Figure 2B). In addition, Figure 2C showed that the CD spectrum of unhydrolyzed *T. molitor* proteins had a major negative trough at around 220 nm, indicating their α-helical structure, whereas this typical α-helix disappeared in the flavourzyme hydrolysates. Moreover, the analysis of the molecular weight distribution of 4-h flavourzyme hydrolysates reveals that the fraction lower than 1000 Da accounted for 67.08% of the total hydrolysate, while the fractions with 1000–3000 Da and >3000 Da accounted for 10.39% and 22.54%, respectively (Figure 2D).

### 3.4. Identification of Peptides of the T. molitor Protein Hydrolysates

**Figure 3:**
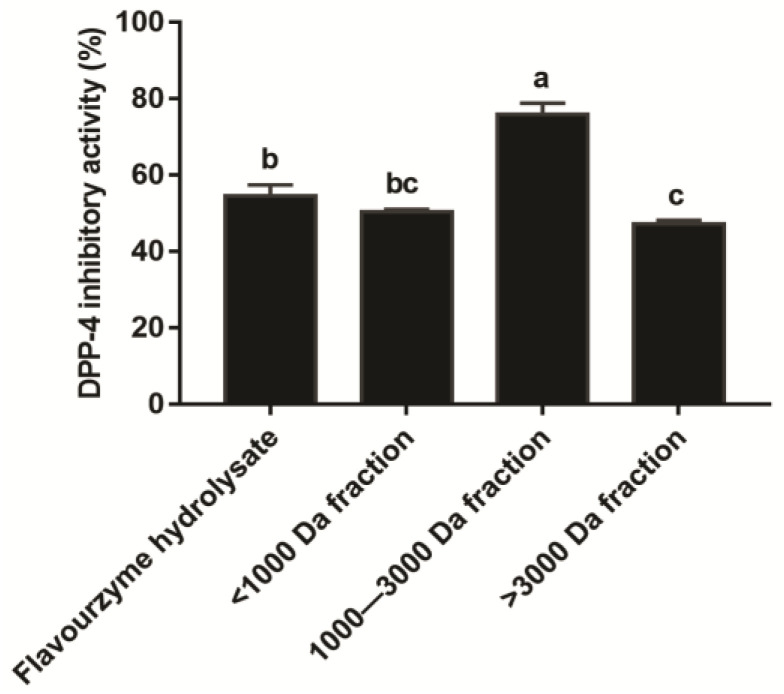
Inhibitory effect of fractions with different molecular weights at a final concentration of 2 mg/mL on DPP-4. Data are shown as the mean ± SD (*n* = 3), and different letters mean significant difference (*p* < 0.05).

In order to identify the peptide from the *T. molitor* protein hydrolysates, we divided the hydrolysates into three fractions according to molecular weight. The results presented in Figure 3 show that, among three fractions, the fraction with 1000 to 3000 Da exhibited the highest inhibition effect on DPP-4, and had higher inhibitory activity against DPP-4 than the 4-h flavourzyme hydrolysates (*p* < 0.05). Therefore, the fraction of 1000–3000 Da was subjected to further analysis for peptide identification. As shown in Table 1, thirteen non-toxic peptides with a PeptideRanker score > 0.60 were identified. It was worth noticing that the PeptideRanker score of peptides APPDGGFWEWGD, LPDQWDWR, NEAYFGFPR, SNGLPFPTRP, and GLQFPTRPNF was higher than 0.80. These peptides were derived from Beta-1, 3-glucanase, C1 family cathepsin L11, Hexamerin 1, Hexamerin 1, and 86 kDa early-staged encapsulation-inducing protein, respectively (Table 1).

### 3.5. The Inhibition of the Synthesized Peptides on DPP-4

**Figure 4:**
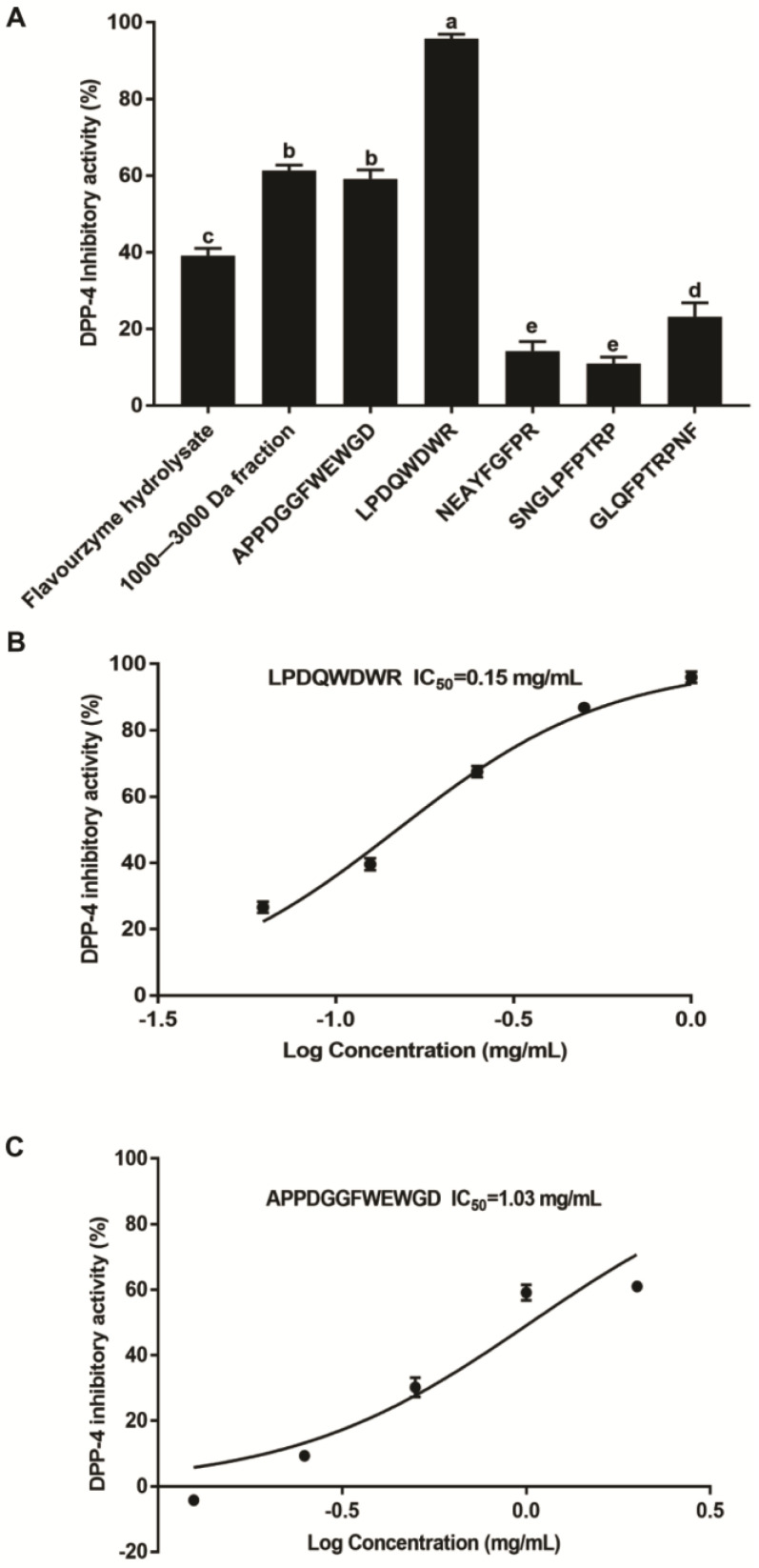
The inhibitory effect of the synthesized peptides on dipeptidyl peptidase-4 (DPP-4) activity. (**A**) Inhibitory effect of the synthesized peptides at a final concentration of 1 mg/mL on DPP-4 activity. (**B**) DPP-4 IC_50_ value of peptide LPDQWDWR against DPP-4 activity. (**C**) IC_50_ value of peptide APPDGGFWEWGD against DPP-4 activity. Data are shown as the mean ± SD (*n* = 3), and different letters mean significant difference (*p* < 0.05).

The peptides APPDGGFWEWGD, LPDQWDWR, NEAYFGFPR, SNGLPFPTRP, and GLQFPTRPNF were synthesized and their activity was evaluated in this study. Among these five peptides, peptide LPDQWDWR showed the highest DPP-4 inhibitory effect, and its inhibitory activity against DPP-4 was markedly higher than that of the flavourzyme hydrolysates and 1000–3000 Da fractions (*p* < 0.05) (Figure 4A). Peptide APPDGGFWEWGD also showed good DPP-4 inhibition, which was not significantly different than that of the 1000–3000 Da fractions (*p* > 0.05). The IC_50_ values of peptides LPDQWDWR and APPDGGFWEWGD against DPP-4 activity were 0.15 and 1.03 mg/mL, respectively (Figure 4B,C). In contrast, peptides NEAYFGFPR, SNGLPFPTRP, and GLQFPTRPNF exhibited weak inhibitory activity against DPP-4 compared to peptides LPDQWDWR and APPDGGFWEWGD (Figure 4A).

### 3.6. Interaction between the Synthesized Peptides and DPP-4 Active Site

**Figure 5:**
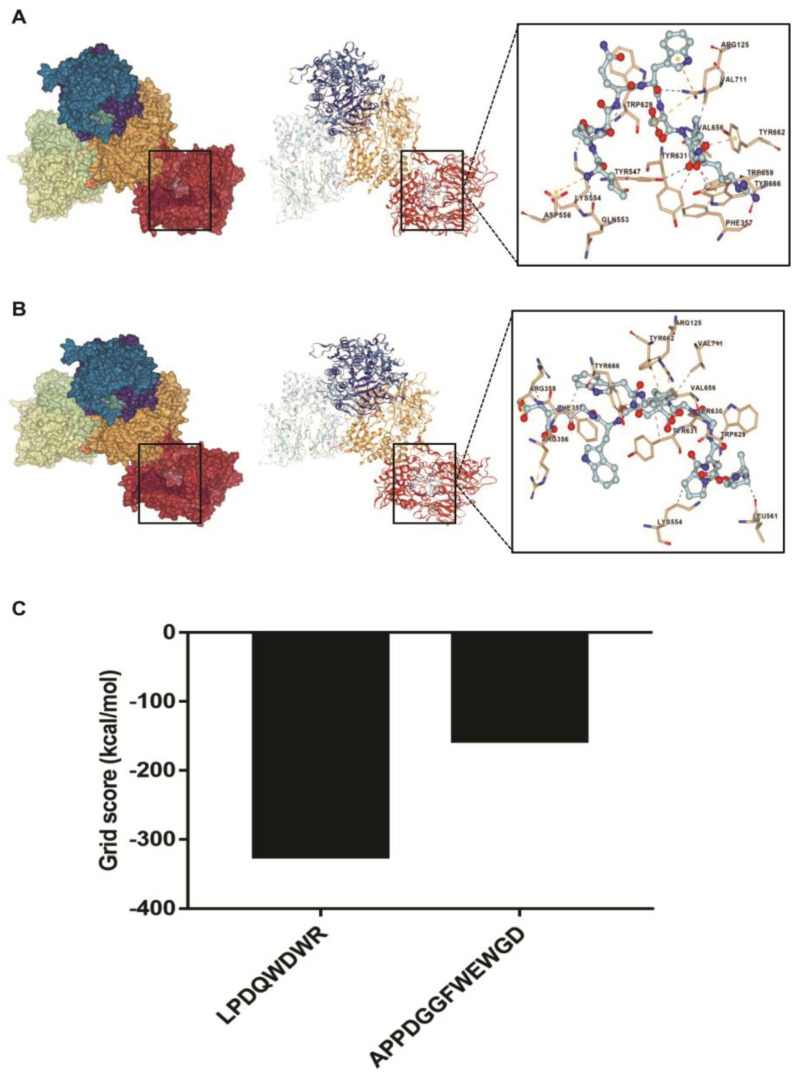
The molecular docking analysis of the synthesized peptides with dipeptidyl peptidase-4 (DPP-4). (**A**) The predicted binding and interaction of peptide LPDQWDWR with DPP-4. (**B**) The predicted binding and interaction of peptide APPDGGFWEWGD with DPP-4. (**C**) The grid score of molecular docking. The yellow dotted line represents salt bridges; the orange dotted line represents π-cation interactions; the green dotted line represents π-π stacking; the blue dotted line represents hydrogen bonding; the grey dotted line represents hydrophobic interactions.

The results presented in Figure 5A and Table 2 show that peptide LPDQWDWR interacted with the Arg125, Asp556, Lys554, Tyr662, Gln553, Tyr547, Val711, Val656, Tyr666, Trp659, Phe357, Tyr631, and Trp629 residues of DPP-4 by hydrophobic interactions, π-cation interactions, π-π stacking, salt bridge formation, and hydrogen bonding. Peptide APPDGGFWEWGD interacted with the Arg125, Arg356, Arg358, Lys554, Tyr662, Leu561, Ser630, Val711, Val656, Tyr666, Phe357, Tyr631, and Trp629 residues of DPP-4 by the above-mentioned interaction types except for π-cation interactions (Figure 5B and Table 2). In addition, as shown in Figure 5C, the grid score of peptide LPDQWDWR against DPP-4 was −324.33 kcal/mol, which was lower than the grid score of peptide APPDGGFWEWGD against DPP-4 (−156.43 kcal/mol).

## 4. Discussion

GLP-1 and GIP are two crucial incretins, which have mainly contributed to the insulin response after meal intake [31]. Suppressing the activity of DPP-4 can increase the level of active incretins, thereby decreasing blood glucose in diabetic patients [32]. Based on the consideration of the adverse effects caused by the chemically synthesized inhibitors, some peptides with inhibitory effects on DPP-4 activity obtained from natural proteins, including milk, egg, grain, edible insects, and aquatic proteins have been widely reported [33,34]. In the current study, the flavourzyme effectively hydrolyzed *T. molitor* proteins, as indicated by the results of the DH, SDS-PAGE analysis, and structural characteristics of flavourzyme hydrolysates. We noticed that the hydrolysis of *T. molitor* protein by flavourzyme was markedly higher than that by the other three enzymes, which was associated with the type of proteases. Alcalase and trypsin are serine endoproteases, and papain belongs to cysteine endoprotease. By contrast, flavourzyme is a mixture including aminopeptidase, carboxypeptidase, and endoprotease [35], indicating that flavourzyme can guarantee extensive hydrolysis of *T. molitor* protein (the maximum DH, 23.25 ± 1.03%). Similarly, Purschke et al. reported that flavourzyme can effectively hydrolyze *Locusta migratoria* protein, and the DH of the 4-h flavourzyme hydrolysates reaches 16.4 ± 0.4% [36]. The DH of *Acheta domesticus* protein hydrolysates generated by 2 h of flavourzyme hydrolysis exceeds 30% [37]. These results indicate that flavourzyme is suitable to be used for the hydrolysis of edible insect proteins. Moreover, the hydrolysates obtained by the 4-h hydrolysis with flavourzyme dose-dependently inhibited the DPP-4 enzyme. The IC_50_ value of the 4-h flavourzyme hydrolysates against DPP-4 activity was 1.64 mg/mL. Consistent with our study, other researchers have reported that the IC_50_ value of *T. molitor* protein hydrolysates against DPP-4 activity ranges from 0.83 to 2.62 mg/mL [23,24]. The IC_50_ value of *T. molitor* protein hydrolysates against DPP-4 activity in these studies is different, likely due to the differences of the enzyme types and hydrolysis conditions used.

It has been reported that the length and size of peptides influence their inhibitory activity against DPP-4. Rivero-Pino et al. obtained and evaluated six fractions with different molecular weight distribution from *T. molitor* protein hydrolysates, and found that the fractions of 500–1600 Da and 1600–3000 Da had a smaller IC_50_ value against DPP-4 in comparison to the original hydrolysates and other fractions [24]. Other researchers also reported that the salmon skin collagen and Antarctic krill-derived peptides below 3000 Da showed a higher inhibitory effect on DPP-4 than other fractions [38,39]. Similar to these results, our study demonstrated that the fraction with 1000–3000 Da had higher DPP-4 inhibition compared with the *T. molitor* protein hydrolysates and other fractions, which indicated that the fraction with 1000–3000 Da has structural features recognized by DPP-4 and thus acts as a substrate for DPP-4. Additionally, the amino acid composition and sequence affect the inhibitory effect of peptides on DPP-4. The occurrence of hydrophobic amino acids in peptides contribute to the binding between peptides and DPP-4 active site, which is crucial to the DPP-4 inhibitory effect of peptides [13,40]. In this study, two novel peptides LPDQWDWR and APPDGGFWEWGD contained 50% hydrophobic residues, and showed excellent inhibitory effects on DPP-4. The DPP-4 inhibitory peptides LPPEHDWR and LPAVTIR identified from *Bombyx mori* protein also contain 50% and 71.43% hydrophobic residues, respectively, and the peptide LPAVTIR with more hydrophobic residues shows more potent inhibition on DPP-4 [34]. Furthermore, the DPP-4 inhibitory peptides with four or more amino acids frequently have leucine/glycine/isoleucine, proline/leucine/lysine, alanine/valine/glycine/proline at positions one, two, and three from their amino terminals, and proline/leucine/arginine at their carboxyl terminals [6]. Among the five synthesized peptides in this study, peptide LPDQWDWR had the typical characteristics of DPP-4 inhibiting peptides, including the leucine and proline at positions 1 and 2 from the amino terminal, and the arginine at the carboxyl-terminal. Similarly, the *Bombyx mori* protein-derived DPP-4 inhibitory peptide LPAVTIR has the leucine, proline, and alanine at positions 1, 2, and 3 from the amino-terminal, and the arginine at the carboxyl-terminal [34], and its IC_50_ value of 0.148 mg/mL against DPP-4 is almost same as the peptide LPDQWDWR identified by our research [34].

The inhibition of peptides against DPP-4 may be dependent on their bindings to the DPP-4 active site. Hydrogen bond and hydrophobic interactions are considered to be critical interaction types [41]. Some peptides derived from food proteins (such as sheep whey and sturgeon skin) can dock into the DPP-4 active site via hydrophobic interactions and hydrogen bonding [42,43]. The Tyr662, His740, Glu205, Ser630, Arg125, Tyr631, Glu206, Tyr547, Phe357, and Tyr666 are important residues of the DPP-4 active site [44]. Our findings showed that peptide LPDQWDWR interacted with the Arg125 residue of the DPP-4 active site by salt bridge formation, π-cation interactions, and hydrogen bonding, and it interacted with the Tyr662, Tyr631, Phe357, and Tyr547 residues by π-π stacking, hydrophobic interactions, hydrophobic interactions, and hydrogen bonding, respectively. Peptide APPDGGFWEWGD interacted with the Arg125, Tyr662, and Ser630 residues of the DPP-4 active site by salt bridge formation, π-π stacking, and hydrogen bonding, respectively, and interacted with the Phe357, Tyr666, and Tyr631 residues by hydrophobic interactions. Taken together, the interaction types and the number of hydrogen bonds of peptide LPDQWDWR with DPP-4 active site were more numerous than those of peptide APPDGGFWEWGD. In addition, peptide LPDQWDWR had a more negative grid score than peptide APPDGGFWEWGD, indicating that peptide LPDQWDWR may form a more stable binding with DPP-4 compared to peptide APPDGGFWEWGD. Thus, peptide LPDQWDWR had a stronger inhibitory activity against DPP-4 than peptide APPDGGFWEWGD.

## 5. Conclusions

Our results demonstrated that the *T. molitor* protein hydrolysates generated by flavourzyme possessed good DPP-4 inhibitory effects. Two novel peptides (LPDQWDWR and APPDGGFWEWGD) identified from the flavourzyme hydrolysates interacted with the DPP-4 active site, thereby potently inhibiting the DPP-4 activity. This study provided evidence for *T. molitor* protein-derived peptides as natural DPP-4 inhibitors, and contributed to the development of *T. molitor*-related functional foods. 

## Figures and Tables

**Table 1:** The screened peptides identified from *Tenebrio molitor* protein hydrolysates generated by flavourzyme hydrolysis for 4 h.

Peptides	Molecular Weight (kDa)	Origin	PeptideRanker	Toxicity
APPDGGFWEWGD	1333.54	Beta-1, 3-glucanase	0.93	Non-Toxin
LPDQWDWR	1115.32	C1 family cathepsin L11	0.88	Non-Toxin
NEAYFGFPR	1100.31	Hexamerin 1	0.87	Non-Toxin
SNGLPFPTRP	1085.36	Hexamerin 1	0.84	Non-Toxin
GLQFPTRPNF	1176.48	86 kDa early-staged encapsulation inducing protein	0.81	Non-Toxin
FPDDVTNPGGKPW	1429.73	Beta-1,3-glucanase	0.76	Non-Toxin
IDDHFLFKEGDRF	1638.98	Arginine kinase	0.70	Non-Toxin
GDYDPDAFNNDIGLIKL	1880.29	Putative serine proteinase	0.69	Non-Toxin
APVIEKPSPGAF	1212.57	Acetylcholinesterase	0.66	Non-Toxin
NGLQFPTRP	1029.29	86 kDa early-staged encapsulation inducing protein	0.66	Non-Toxin
IGGGDANAGEFPF	1251.50	Putative serine proteinase	0.64	Non-Toxin
YPFWMSGEEFNLK	1648.05	Hexamerin 2	0.61	Non-Toxin
APDYEEANGKGVIIF	1623.01	Glutathione S-transferase delta	0.60	Non-Toxin

**Table 2:** The interaction types and amino acid residues of the synthesized peptides with dipeptidyl peptidase-4.

Peptides	Salt Bridges	π-Cation Interactions	π-π Stacking	Hydrogen Bonding	Hydrophobic Interactions
LPDQWDWR	Arg125, Asp556, Lys554	Arg125	Tyr662	Arg125, Gln553, Tyr547	Val711, Val656, Tyr666, Trp659, Phe357, Tyr631, Trp629
APPDGGFWEWGD	Arg125, Arg356, Arg358, Lys554	/	Tyr662	Leu561, Ser630	Val711, Val656, Tyr666, Arg358, Phe357, Tyr631, Trp629, Lys554

## Data Availability

Not applicable.
